# Use of the School Setting During the Summer Holidays: Mixed‐Methods Evaluation of Food and Fun Clubs in Wales

**DOI:** 10.1111/josh.12824

**Published:** 2019-07-28

**Authors:** Kelly Morgan, Linda McConnon, Jordan Van Godwin, Jemma Hawkins, Amy Bond, Adam Fletcher

**Affiliations:** ^1^ DECIPHer, School of Social Sciences Cardiff University, 1‐3 Museum Place Cardiff CF10 3BD UK; ^2^ Y Lab, School of Social Sciences Cardiff University, 56 Park Place Cardiff CF10 3AT UK

**Keywords:** school setting, policy, physical activity, diet, accelerometry, healthy eating

## Abstract

**OBJECTIVE:**

School summer holiday clubs in deprived areas of Wales were evaluated to examine opportunities for healthy eating and physical activity and explore delivery processes.

**METHODS:**

Ten Food and Fun clubs participated in 2016. Quantitative data (child and parent surveys; N = 196, N = 84) assessed the opportunity to provide children with breakfast and lunch. A sub‐sample of children wore an accelerometer (N = 41) to evaluate the opportunity for achieving 1‐hour of moderate to vigorous activity (MVPA) at club. Features of successful club delivery were identified through; focus groups (child and parent; N = 74, N = 69) and interviews (staff/volunteer; N = 32).

**RESULTS:**

Opportunities for healthy eating were delivered with high fidelity: 86% of children reported breakfast consumption and 75% eating a healthy lunch. On club days, children reported consuming fewer sugary snacks (66%), fewer sugary drinks (81%), and more fruits and vegetables (67%). About 71% of children achieved the recommended MVPA at club, with children engaging in more MVPA (+17 minutes/day, p < .01) on average compared to non‐club days. Successful delivery processes were: use of school facilities and staff; flexible partnership‐working; and whole family involvement.

**CONCLUSIONS:**

Schools appear to provide a suitable setting for the delivery of healthy eating and physical activity opportunities during school summer holidays.

A large number of children are currently receiving inadequate food for a healthy diet, due to the growing problem of food insecurity.^1^, ^2^ Often constructed by 4 dimensions; food quantity, food quality, feelings of deprivation and disrupted eating,^3^ earlier figures revealed that 10% of individuals aged 15 and older are affected by food insecurity in the United Kingdom.^4^ As such, food insecurity is a critical social policy issue in the United Kingdom, with an increasing number of families reliant on food aid.^5^ Among children, food insecurity has been associated with lower fruit and vegetable intake,^6^, ^7^ a lack of physical activity^8^, ^9^ and poor health and educational outcomes.^10^ Children experiencing food insecurity may also be at a greater risk of becoming overweight or obese.^11^, ^12^ These risks and behaviors cluster among children living in poorer households and are patterned socio‐geographically, with widening inequalities emerging over the past 20 years in terms of childhood nutrition and obesity.^13^, ^14^

The school environment has been shown to play an important role in promoting healthier diets among children.^15^ Accounting for more than one‐third of children's daily caloric intake during the year,^16^ schools provide an opportune place to implement policies that promote healthy food choices within a familiar and accessible environment. A current example is the provision of school lunches in Wales, with 18.4% of primary school pupils at present in receipt of free school lunches in Wales.^17^ Another universal approach to reduce child health inequalities now adopted in the United Kingdom is the provision of free school breakfasts for all primary school‐aged children (aged 5‐11 years). However, withdrawal of both schemes during the 6‐week summer holidays has implications in deprived communities, with families having to source up to 60 extra meals per child. A recent small‐scale survey of UK teachers revealed concerns that children do not get enough to eat during the school holidays.^18^ As well as school holiday food poverty,^19^, ^20^ the quality of children's diets is also an area of concern—and these concerns are not limited to the United Kingdom. Findings from a large US study showed that children generally consumed more sugary beverages and fewer vegetables during school holidays, with findings consistent across all ages and household income levels.^21^ Limited research has attempted to deduce whether school food policies and practices are influential on children's overall eating patterns outside of the school environment.^22^

Sedentary behavior during the school holidays is also a worldwide concern, with only 15% of 11‐ to 15‐year olds currently managing an hour of daily activity^23^ and further declines noted during school holidays.^24^ Findings from a UK‐based study showed decreased physical fitness and increased weight gain among 8‐ to 9‐year olds following the summer holidays.^25^ Attributing these findings to inadequate physical activity levels, the authors concluded that greater activity provision was necessary during the holiday period, echoing recent policy recommendations.^26^

Concerns about child health and well‐being during the school holidays have led to the development and implementation of new summer holiday initiatives aiming to increase access to food and physical activity provision, especially in low‐income communities.^18^, ^20^, ^27^ With federally funded summer programs running over the past 25 years, much of the evidence on holiday initiatives has been conducted in the United States. A recent evaluation of US programs highlighted their effectiveness but also the importance of assuring nutritional standards and using existing resources,^28^ however, the transferability of such programs to other settings remains uncertain with little evaluation outside the United States. There is also limited evidence about the wider impact of food provision from holiday initiatives (eg, do meals provided displace unhealthier foods otherwise consumed or substitute meals which would otherwise be missing). Minimal research has assessed the accumulation of physical activity within holiday initiatives, with findings limited to a few US‐based studies.^29^, ^30^

As of yet, no formal evaluation of a UK‐based summer holiday club has been carried out. One such initiative based in the United Kingdom, is Food and Fun clubs, implemented within schools in areas of high deprivation across Wales. Developed and piloted in 2015 within one local authority, Food and Fun is a multi‐agency project providing healthy meals (adhering to school nutrition standards), nutrition skills and physical activities during the summer holiday period. The aims of this study were to (1) investigate the healthy eating and physical activity opportunities provided at Food and Fun holiday clubs and (2) explore the barriers and facilitators to delivering these clubs.

## METHODS

### Settings

During the school summer holiday period of 2016, 10 schools (6 primary school and 4 secondary school sites) from 5 local authorities in Wales delivered a Food and Fun club and agreed to participate within the evaluation study. Five of the 6 primary school sites invited pupils attending their own school while the remaining primary school site invited Year 5 and 6 children (age 9‐11) from various local primary schools as well as their own pupils. Of the secondary school sites, 2 schools provided open access clubs for all local children; one school invited its own Year 8 and 9 students (age 12‐14); and one school provided a “transition” program for its incoming Year 7 students (age 10‐11). Recruitment strategies varied by school, with a mixture of targeted (eg, only children meeting pre‐specified criteria were invited) and inclusive approaches (eg, all children in year groups 3‐5 invited) being used.

### Participants

For each child invited to attend a Food and Fun club, parents and guardians were provided with study information and the opportunity to part or fully opt their child out of the study. Parents were also offered the opportunity to participate in the study through completing a survey and/or taking part in a focus group. Separate opt‐in consent was obtained for collecting objective physical activity data from children. Child assent was also collected prior to each component of data collection.

Across the 10 schools, club registers revealed that 323 children attended at least 1 day, with the majority (78%) of children aged 7‐11 years (range 3‐14 years). Attendance records showed that 53% of children attended at least 6 of the 12 days provided, with 13.6% attending all 12 days. There were 300 occurrences in which a parent attended a family day at club to eat lunch with their child. The proportion of children eligible for free school meals across the school sites ranged from 22% to 57% (mean 34.9%).

### Instruments

#### *Surveys*

Children were provided with a 22‐point survey to explore their views and experiences of club attendance, daily activities and eating habits during non‐club days, and home environment. Parents or a guardian were provided with a similarly designed 41‐point survey, which included extra sections covering demographics, financial circumstances and food availability and diet at home. Dietary questions were used to assess intervention fidelity of clubs providing 2 nutritious meals a day.

#### *Accelerometry*

To assess the proportion of children achieving 60 minutes of moderate to vigorous activity (MVPA) on club days, a sub‐sample of children were asked to wear an ActiGraph GT3x+ (ActiGraph LLC, Pensacola, FL) accelerometer for 7 consecutive days. The monitor was attached to a belt and placed around the right hip of each child, with instructions to remove the device only during hours of bathing or sleeping.

#### *Focus groups*

Children actively participated in focus groups to voice their opinions of the Food and Fun club. With use of playful and artistic methods (making posters, picking ideation cards and drawing plates of food), children discussed a typical lunch both within the home and club environment, and their routine activities when the club is closed. Parent focus groups explored the challenges to providing food and fun activities during the summer holidays, beneficial impacts of the club and their recommendations for the future.

#### *Interviews*

With use of an interview topic guide, a variety of staff members and volunteers at each club were asked to reflect on their job role, feedback comments from children and parents, the potential benefits of the project and their experiences of working at a club.

### Procedure

A researcher attended the last club day of each week (maximum of 4 visits), coinciding with a family club day (parents and siblings were invited to club to enjoy a free lunch). During the first week, a sub‐sample of children were fitted with initialized accelerometers and later provided with a £10 gift voucher upon return of the device. During the second week, children and parents completed surveys during club time and focus groups and interviews were conducted during the third or fourth week. A £10 gift voucher was provided for each parent taking part in a focus group.

### Data Analysis

Descriptive statistics of survey data were calculated to describe the sample and examine opportunities for healthy eating and physical activity. Raw accelerometer data were processed with the ActiLife software (version 6; ActiGraph LLC). Periods of ≥60 minutes of zero values were defined as accelerometer “non‐wear” time and discarded. Participants were included in the analysis if they provided ≥3 days of data (including at least one; club‐, non‐club‐, and weekend day) with at least 500 minutes of data between 6 am and 11 pm. A cut‐point of ≥2296 counts per minute^31^ was used to identify mean minutes of MVPA on club, non‐club, and weekend days. *t* tests were used to determine if children who provided 3 days of valid data differed to those that did not provide valid data on the following characteristics; sex, year group, and material deprivation. Significance levels were set at p ≤ .05. Paired sample *t* tests were used to examine differences between accelerometer data on club days and non‐club days. To test for equality of proportions meeting the recommended daily activity levels across the 3 days a Cochran's Q test^32^ was carried out and McNemar's tests employed for post hoc group comparisons.

Focus groups and interviews were conducted in person, audio‐recorded and transcribed verbatim, with NVivo 11 software (NVivo qualitative data analysis Software; QSR International Pty Ltd. Version 11, Melbourne, Australia) used for all analyzes. Two members of the research team reviewed the transcripts, after which the following method was adopted: line by line open coding (descriptive labeling), axial coding (clustering relationships, links and associations), and selective coding (exploring key codes and variables).

## RESULTS

### Demographics

A total of 196 children and 84 parents/guardians completed a survey (Table 1), with a sub‐sample of 48 children providing accelerometer data. The child material deprivation index measure^33^ revealed that approximately 47% of children surveyed were classified as either deprived (18.4%), very deprived (21.1%), or severely deprived (7.5%). Focus groups were carried out with 74 children and 69 parents. In total, 32 Food and Fun staff and volunteers participated in a semi‐structured interview during the running of the club. Of those interviewed, 19% were male and staff comprised; school, catering, and external members.

**Table 1:** Demographics of Children, Parents and Staff†

Demographic	N (%)
Children	
Key stage	
1 (Years 1‐2)	20 (10.4)
2 (Years 3‐6)	129 (67.2)
3 (Years 7‐9)	41 (21.4)
4 (Years 10‐11)	2 (1.0)
Sex	
Boys	90 (45.9)
Girls	106 (54.1)
Race/ethnicity	
White British	144 (79.1)
Asian	19 (10.4)
Black	6 (3.3)
Mixed	9 (5)
Other	4 (2.2)
Material deprivation	
Not deprived	98 (53)
Deprived	34 (18.4)
Very deprived	39 (21.1)
Severely deprived	14 (7.5)
Parent/guardian	
Relation to child	
Parent	77 (91.7)
Grandparent	4 (4.8)
Other	3 (3.6)
Age	
18‐24	2 (2.4)
25‐34	35 (41.7)
35‐44	34 (40.5)
45‐54	8 (9.5)
55+	5 (6)
Employment status	
Employed	41 (49.4)
Homemaker	25 (30.1)
Unemployed	14 (16.9)
Education	3 (3.6)
Education	
School	31 (37.8)
BTEC/vocational	31 (37.8)
Higher	9 (11.0)
None	11 (13.4)
Staff and volunteers	
Male	6 (18.8)
Female	26 (81.2)

† Participants who completed a survey.

### Healthy Eating Opportunities

The goal of implementation monitoring was to assess the opportunities for healthy eating and achieving 1‐hour of physical activity while attending a Food and Fun club. Dose delivered and fidelity were captured through child and adult surveys and accelerometer data.

Overall, 86% (N = 166) of children surveyed reported eating breakfast and 75% (N = 147) a healthy lunch on a club day. Of those not eating breakfast at club, 12% (N = 24) reported eating breakfast at home before going to club. When asked to respond “yes (including ‘sometimes’)” or “no” to statements regarding food intake at club compared to food intake at home, 67% (N = 131), 66% (N = 129), and 81% (N = 159) reported “yes” to consuming; more fruit and vegetables, fewer sugary snacks and fewer sugary and fizzy drinks, respectively, at club. One in 5 children (N = 39) reported that they do not get enough to eat at home because they run out of food and 28% (N = 55) reported feeling hungry at home a lot.

Among parental survey responses, 36% (N = 27) and 11% (N = 7) reported that their child ate unhealthy foods or not enough food, respectively, when the club was not open. One‐fifth of parents (N = 17) reported meal skipping so that their child would have food to eat. With the running of the club, 53% (N = 44) stated that food lasted longer at home and 36% (N = 16) reported fewer meal skipping than they usually would during holiday months.

### Physical Activity Opportunities

Valid accelerometer data were available for 24% (N = 48) of surveyed children, with an equal split of both sexes. No differences (sex, age, or deprivation) were observed between the sub‐sample of children provided with an accelerometer and the wider study population. Of the 78 accelerometers given out, 3 were unreturned, 10 had faulty data and 17 did not meet the inclusion criteria. We found that sex and material deprivation were predictive of data validity, with boys and those reporting greater material deprivation scores more likely to provide insufficient data (p ≤ .05 on both accounts). The goal of the intervention was for children to achieve the recommended 60 minutes of MVPA at club. Accelerometer data (Table 2) revealed that this goal was achieved by 71% of children, in comparison to 48% on non‐club weekdays and 55% on weekends, with a significant difference observed between club‐ and non‐club days. Parents also reported that the club helped their child to be more active over the summer holidays (93%).

**Table 2:** Accelerometry Data on Club, Non‐club and Weekend Days (Average Minutes)

	Club Day (N = 48)†	Non‐club Weekday (N = 48)	Weekend Day (N = 44)
Accelerometer Data (minutes)	Median (IQR)	Median (IQR)	Median (IQR)
Total valid wear time	1455.17 (1336.24‐1863.75)	808.17 (685.59‐1008.75)	1227.36 (802.25‐1437)
Average valid	718.92 (649.38‐772.86)	724.88 (618.67‐824.17)	715.08 (621.38‐759.96)
Average MVPA	81.75 (56.75‐96.79)	58.27 (43.63‐81.75)**	44.08 (27.71‐78.08)*
Achieved recommended MVPA (%)	70.8	47.9*	54.5

* p < .05.

** p < .01.

IQR, interquartile range (25th‐75th).

† Reference group.

### Barriers and Facilitators to Delivering Food and Fun Holiday Clubs

**Figure 1:**
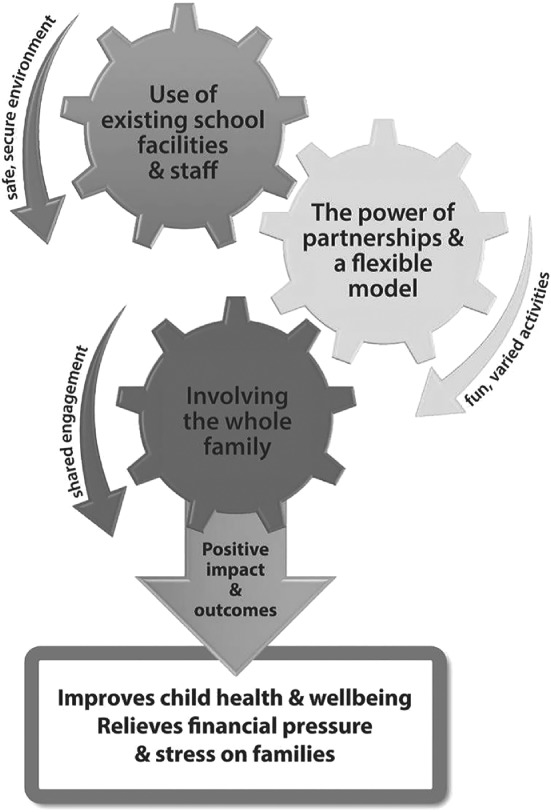
Working Elements of Food and Fun Clubs

Themes within 3 domains emerged from the interviews and focus group data: (1) use of existing school facilities and staff, (2) the power of partnerships and a flexible model, and (3) involving the whole family. Table 3 provides illustrative quotes within each domain and Figure 1 depicts how each domain within a Food and Fun club can have a positive impact on child health and well‐being.

**Table 3:** In‐depth Interview Themes and Illustrative Quotes

Thematic Domain	Staff and Volunteers	Parents	Child
Use of existing school facilities and staff	“I enjoyed the interaction with parents as well. Hopefully trying to break down a few barriers and let them know that the school is a friendly place and not to worry”—School staff “Children here should be familiar with all of the meals so it's nothing new that they're trying. It's things that they have during their school lunches, which is good”—School catering staff “Six weeks of this year it's not used, … it's shut down… now it's being utilized and it's not going to waste”—School staff “Everything's been just the same, it's like a normal working week for us”—School catering staff	“I know in the back of my head, my kids are safe, you know the school, you know your way around here, you know you're safe” “You get to speak to the teachers” “I think it makes it less scary for September being based in the school” “People that she knows, I want her to be with people that she knows”	“I like the teachers and the people in holiday club” “I'm not missing school as much” “Teachers, they are kind” “When you get used to it, you're more happy and you do more things, try more things” “Saves you paying for food and you don't know if you are going to like it or not”
The power of partnerships and a flexible model	“It wasn't such a structured program, if it was a nice day, [the leader] would stop the session and then take them out and maybe do a bit more keep fit or rounders”—Local Authority Principal Catering Officer “Sport Cardiff were showing the staff how to use those resources in different ways”—Senior Sport Neighborhood Coordinator “The range of providers and the activities was fantastic, really good. So the mornings were great because it was really sporty, really active, and then the afternoon sessions were more creative and really nobody was bored”—School staff	“The variety they put on is fantastic!” “He used to go to a play scheme, but he's gone too old for that” “Anything he's made we have had to put up. The dream catcher is above the bed. The roly‐poly bird is on his bookshelf so it's by all his books. The parrot is hanging from the ceiling”	“I like the stuff in Food and Fun, different things because we don't do that normally” “So many things you can do, there are activities for everyone” “We like the session when you taste the food when you were blindfolded” “I like the sport, especially football, rugby, cricket and rounders and basketball” “We get to do things we haven't done before”
Involving the whole family	“Invite the parents for the whole week as well to bring them in”—School catering staff “There are parents who don't want to come into school, who don't want to attend. So I think anything positive we can do to try to address that is something that is important for the school”—School staff “It's been nice to see the parents coming in because there's one child who said I wish my mum would come in because she does things with my older sister and not me. So the second week mum has come in and had lunch so it was nice to see mum, dad and her just having their alone time”—School staff	“It is nice that the parents can come as well and get involved with the kids and see what they have done and what food they have eaten throughout the week” “Speaking to an adult is different from speaking to a child, it is nice to speak to an adult as well and see the kids sitting down with their parents enjoying it” “That's why it's been nice for my other 2 coming to the other clubs because I've had more time with my youngest boy” “I did like the quiz… you know when the parents interacted with the children when they did that… it was brilliant, because everyone was involved”	“I like parent days because you can eat with them and tell your family what you've been doing” “Parents will be friends as well” “I just think it helps them knowing my friends that I've met here… maybe I did go [sic] to their house sometime or they could come to my house sometimes” “They, [siblings] usually hurt each other and me, so if they come here, they might learn to be nice”

#### *Use of existing school facilities and staff*

A common theme across all staff interviews was the benefit of using the school facilities and the recruitment of existing staff members. Positive themes related to the use of high‐quality, trained staff, staff familiarity with children and families attending, and the opportunity for continual learning in future term times (eg, delivering nutrition education or sports activities). Echoing staff, parents highlighted the reassurance felt of using a safe and secure environment with trusted staff running the club.

Exploiting the use of existing school facilities, in particular kitchen facilities and sports equipment was also highlighted, with catering staff emphasizing the ease of maintaining usual practice throughout the summer holidays, when facilities usually remained unused. Some staff perceived that hosting a Food and Fun club within the school setting was conducive to geographical targeting rather than other approaches which may lead to the labeling or stigmatizing of those families invited.

A number of staff perceived the ability to create an environment enabling child autonomy and agency as an important aspect for catering to children's needs. Specific examples provided by staff included the style of authority adopted, for example, a play‐worker verses teacher approach, and adopting fun activities. Additionally, staff explicitly stated the need to avoid standard classroom activities, with some staff reporting a similarity between nutrition sessions and standard school lessons. The dislike of such instances was also expressed by children during focus groups.

#### *The power of partnerships and a flexible model*

The ability to tailor the Food and Fun model at a local community level was found to be important. While all staff reported the provision of the standard Food and Fun daily components, 2 positive themes related directly to the running of the club: partnership working and flexibility of the model.

Staff discussed the value of drawing on local assets and engaging with external organizations, relaying how this process revealed alternative ways of using existing school resources and enhanced the professional development of staff. Children and parents remarked on the variety and novelty of the activities delivered by both staff and volunteers at the club, with children stating that they had never had these opportunities before. While expressing their enjoyment of having a full‐timetable of activities, children also highlighted the enjoyment of undertaking free play where it was available. Alongside positive reports of wide community engagement, some parents noted the number of competing free schemes within the community. This was highlighted as a barrier for child attendance alongside inadequate advertising and a lack of transport to club.

The importance of the model's flexibility was prominent in both staff and parental interviews. Staff perceived flexibility as instrumental to the recruitment and retention of quality staff and to the general running of the day, reporting ownership over daily content and delivery in achieving the club's aims and objectives. Parents positively viewed club opening times, stating that flexibility supported attendance. For example, running over a series of weeks allowed a trial period if their child was anxious or unsettled, a concern noted by parents, or attending for only some days if they had prior commitments.

#### *Involving the whole family*

Children, parents and other family members expressed their enjoyment of sharing mealtimes together, with opportunities to share experiences and also socialize with other parents and community members. Staff and volunteers emphasized the value of a whole family approach, in order to achieve the club's aims and ethos and to engage hard to reach families. At clubs where this was particularly evident, staff and local agencies welcomed the opportunity to build new relationships with families and exchange information. On the other hand, some parents deemed the specific targeting of families as stigmatizing and did not feel this was appropriate for a summer holiday initiative.

Parents liked having direct, open access to school staff and the school environment. Many parents said this gave them fresh insights, new perspectives and a deeper understanding of how their children behaved and interacted with others in school. In instances where existing school staff and volunteers were employed to deliver the club, familiarity was noted as a way of appeasing family concerns of attending club and building rapport, highlighted during parent focus groups.

## DISCUSSION

This study is the first to evaluate opportunities for summer holiday provision within the school‐setting in the United Kingdom. Findings from the 10 school sites highlighted that opportunities for children to eat 2 healthy meals a day and meet physical activity guidelines can be provided during the summer holiday period.

### Healthy Eating

In our sample, a lack of food availability during usual school holiday practices was evident. Nearly one‐third of children reported insufficient food and feeling hungry a lot at home and one‐fifth of parents reported meal skipping in order to provide food for their child. In the context of food provision, positive findings emerged from attending a Food and Fun club, with over half of parents reporting that the club enabled them to make food last longer and over one‐third having to skip fewer meals than they usually would during holiday months. The club aimed to provide children with 2 healthy meals a day, of which the majority of children reported consuming breakfast and three‐fourths a healthy lunch.

We found reports of healthier dietary intake among children at club, with fewer sugary snacks, fewer sugary drinks, and a greater intake of fruits and vegetables compared to diets at home. Findings from the current study suggest the potential for a dual mechanism, whereby food provision at club is not only addressing the lack of food availability during the school holidays but also replacing unhealthy food items with healthier alternatives. The importance of such mechanism has been stressed previously,^34^ being noted as *a must* for interventions aiming to address social inequalities.

Our results are consistent with prior findings from the United States that children are more likely to consume healthier diets during the school holidays if they are involved in a structured environment (ie, summer camp).^29^ On the contrary, one US‐based study found a high intake of processed foods and inadequate vegetables among children attending a summer camp, with authors concluding that the nutritional quality of food could be improved.^35^ While using direct, valid observation methods, the authors acknowledged their inability to comment on foods consumed within the home environment. Our findings are also encouraging in light of recent National Health and Nutrition Examination Survey data, which demonstrates poorer diets (ie, more sugar and less fruit and vegetables) during summer breaks when compared to children's diets during school terms.^21^

### Physical Activity

To date a limited number of studies have directly examined levels of child activity during school holidays with existing reports largely concerning child fitness levels.^25^ This study is the first to objectively gather data on children's usual activity levels over the school holidays and days at a school holiday club. We found that club attendance provided the opportunity to achieve the recommended 1‐hour of MVPA compared to non‐club days (including weekends), with parents also emphasizing that the club helped their child to be more active over the holidays. Our findings are consistent with Tovar et al.,^29^ who found greater activity levels among children attending an American summer camp compared to activity levels of children in parent care. Similarly, Baker et al.^30^ recently concluded that attendance at a 6‐day US summer camp helped children achieve the recommended daily MVPA, however, the authors were unable to determine if children were more active at camp compared to usual practices due to a limited study design.

The Food and Fun initiative consists of a 1‐hour daily physical activity session delivered by a member of staff or external sporting agency. Our findings indicated that the dose of physical activity was inconsistent, with over a quarter of children not achieving the recommended 1‐hour of MVPA on club days. Even though children were more likely to be active while attending club and the majority reported breathlessness and sweating during activities, our findings question the intensity and structure of sessions as not all children achieved the recommended levels on club days. Further research with a larger sample and use of observations would help identify the content, delivery, and involvement of children within these sessions.

### Implementing School Holiday Clubs

The results provide new evidence regarding the use of the school setting for providing holiday provision within the UK‐setting. We identified 3 main elements underpinning the delivery of a Food and Fun club: use of existing facilities, whole family approach and the power of partnerships and a flexible model. As such, the Food and Fun initiative demonstrates a working example of current policy recommendations,^36^ which call for adjoining school facilities and community services in order to enhance child outcomes. Staff, volunteers, and parents all perceived the school setting as conducive to the running of the club, emphasizing the model's simplicity of using existing resources and providing a familiar environment for staff, children, and parents alike. Findings highlight important considerations when recruiting children and families. These results are positive in light of earlier findings which suggest that children may be more likely to gain weight over the school holidays when in a relatively unstructured environment.^37^ To our knowledge, no study to date has examined the use of the school setting for the delivery of school holiday clubs, highlighting the novel approach adopted by Food and Fun clubs and the possibilities for future initiatives. Although the results warrant future research using larger samples, findings suggest that Food and Fun clubs are one approach for utilizing existing resources to aid children in achieving healthy behaviors during the school holidays.

### Limitations

As a small‐scale evaluation with a cross‐sectional design, our findings are subject to several limitations including small sample size, selection and reporting bias, and limited generalizability. Measures of healthy eating opportunities were obtained from self‐reports and not direct observations, however, it is important to acknowledge that Food and Fun clubs benefit from school regulations with all meals adhering to Healthy School nutritional standards. The use of accelerometry increased the quality of activity data collected yet problems encountered such as unreturned devices and lack of wear time significantly reduced the anticipated data pool, often common reporting of accelerometry use within studies.^38^ Nonetheless, the use of a mixed‐methods approach to triangulate data and enhance our understanding of the potential impacts and delivery mechanisms was a particular strength.

### Conclusions

This study represents an important early step in our understanding of how the school environment can aid the delivery of initiatives, such as Food and Fun clubs, during the school summer holidays. The school environment appeared to play a pivotal role in facilitating child and family attendance, with factors such as familiarity, safety, and resources influencing outcomes. Findings regarding the dose and fidelity of the intervention highlight areas that require further attention if any future full‐scale evaluation is to be carried out.

## IMPLICATIONS FOR SCHOOL HEALTH

Previous research suggests that food insecurity is a growing issue for school‐aged children and is linked to various adverse health and educational outcomes.^10^, ^11^, ^12^ Schools may be able to help protect against such adverse outcomes by providing summer holiday initiatives on the school premises, with opportunities for consumption of a healthy breakfast and lunch as well as enriching activities. Initiatives based in the United States have shown promise for impacting on school health^28^ and this study has extended these findings to the UK context, with preliminary evidence for addressing lack of food availability, consumption of unhealthy foods, and achieving recommended daily physical activity levels during the school summer holidays.

Our findings offer some insight into delivery approaches to such initiatives that may be particularly acceptable and feasible to students and their parents, as well as to the staff that deliver them. For example, engaging existing school staff in such initiatives can be important for ensuring high‐quality delivery and for capitalizing on existing relationships and familiarity between staff, students, and parents. Employing external staff to run such initiatives may not be as well received. While our data suggest that initiatives should be coordinated and overseen by existing school staff, engaging external community organizations to support delivery of specific activities can help enhance the initiative; providing children with opportunities to try new experiences as well as creating links with community‐based opportunities for future engagement. In addition, regarding the content and focus of activities provided during such initiatives, our findings suggest that activities should be substantially different from typical classroom lessons, with an emphasis on fun and enjoyment.

In terms of the practical implications on a day‐to‐day basis, schools should aim to maximize attendance through ensuring the initiative is accessible with transportation options. Schools should also seek to engage students' families on a weekly basis. This would allow further opportunities to maximize child attendance and provide time to impact positively on relationships between schools and families, and between families themselves.

### Human Subjects Approval Statement

The School of Social Sciences Research Ethics Committee at Cardiff University (SREC/1882) approved this study.
